# Addressing neglected tropical diseases in Africa: A gender perspective

**DOI:** 10.1002/hsr2.1726

**Published:** 2023-11-16

**Authors:** Deborah O. Shomuyiwa, Nsikakabasi S. George, Blessing A. Sunday, Faith O. Omotayo, Martha Mwaba, Success C. David, Maxencia Nabiryo, Yonah Yangaza

**Affiliations:** ^1^ Faculty of Pharmacy University of Lagos Lagos Nigeria; ^2^ School of Heath and Related Research The University of Sheffield Sheffield UK; ^3^ École des hautes études en santé publique Rennes France; ^4^ Faculty of Pharmacy University of Uyo Uyo Nigeria; ^5^ Faculty of Pharmaceutical Sciences University of Nigeria Nsukka Enugu Nigeria; ^6^ Department of Public and Community Health Science University of the People California USA; ^7^ Ministry of Health Lusaka Zambia; ^8^ Integrated Community Health Initiative Organization Kampala Uganda; ^9^ Makerere University School of Public Health Kampala Uganda; ^10^ One Health Society Dar es salaam Tanzania

**Keywords:** Africa, epidemiology, gender, health equity, neglected tropical diseases

## Abstract

This article delves into the interplay of neglected tropical diseases (NTDs) and Sustainable Development Goals (SDGs) within Africa, spotlighting gender disparities in NTD programs. NTDs, impacting marginalized communities, impose considerable physical, mental, and social burdens. The article underscores NTDs as equity markers for SDGs, spotlighting gender‐based imbalances in disease susceptibility, treatment accessibility, and health‐seeking tendencies. Gender's influence on NTD risks is elucidated, emphasizing the heightened susceptibility of women due to socioeconomic constraints, cultural dynamics, and gender norms. The article also highlights the absence of gender considerations in NTD programs, advocating for gender‐integrated strategies, enhanced data collection, and collaborative partnerships to rectify these inequities. By embracing a gender‐equity approach, the article underscores the necessity of gender‐balanced NTD efforts for comprehensive health, sustainable development, and gender parity, demanding cohesive actions across sectors.

## 1 INTRODUCTION

The foundation of the Sustainable Development Goals (SDGs) centers around the mantra of “leave no one behind.” This mantra embodies the collective commitment to forge a better and more sustainable future for all individuals by 2030.^1^ This principle holds immense significance in the context of addressing neglected tropical diseases (NTDs). NTDs, a cluster of 20 diseases primarily associated with impoverished conditions, disproportionately affect the most vulnerable and marginalized communities.^2^ These diseases impose severe physical, mental, and social burdens, further hampering economic growth and potential. With a staggering global impact affecting over 1 billion people, NTDs form an integral facet of SDG 3, which aims to “ensure healthy lives and promote wellbeing for all ages.”^2^

With formal recognition as a target for global action towards the SDGs, global efforts towards the eradication of NTDs include WHO's development of a strategic roadmap for the prevention and control of NTDs as well as cross‐cutting targets to facilitate change in operating culture as well as accountability in global efforts toward NTDs in 2021.^3^ The policy document replacing the 2012 roadmap aims to drive a 90% reduction in NTD cases requiring treatment and eradication of at least one NTD in 100 countries.^3^ This heralded a wide range of innovative management strategies, including large‐scale preventive treatments, integrated vector management, improved access to water, sanitation, and hygiene, defined public health, and intensified disease management.^4^ Difficulties in putting these measures into action include a lack of suitable tools and technology for proper implementation, limited reach among at‐risk populations, inadequate surveillance systems, and ineffective focus on targeted interventions.^5^

NTDs are pivotal indicators for the SDGs, offering insights into the scope of inequality in disease impact and enabling the assessment of disparities.^6^ These disparities extend to the realm of universal health coverage and equitable access to quality healthcare services, influenced by factors including geography and notably, gender.^7^ Gender, identified as a significant source of inequity, plays a crucial role. Gender dynamics and gender‐equitable access to NTD interventions are vital considerations. SDG 5, centered on equity and justice, seeks gender equality through eradicating discrimination and empowering all women, with community‐led medicine distribution and sanitation integration underscoring women's pivotal role.^8^ Gender equity is integral to just societies and SDGs attainment, necessitating the resolution of gender‐based disparities in NTD perception, health‐seeking behavior, care access, and disease management. While NTDs burden both genders, specific diseases disproportionately affect girls and women, underscoring the need for gender‐sensitive approaches.^4^ This article aims to spotlight and address gender disparities in African NTD programs, emphasizing the need for gender‐inclusive strategies to ensure equitable healthcare access, and aligning with SDGs aspirations.

## 2 IMPACT OF SEX AND GENDER ON NTDS RISKS AND OUTCOMES

The gendered division of household tasks, pregnancy, and health‐seeking barriers such as diminished economic power, illiteracy, and social behavior such as stigma and discrimination increase girls' and women's NTD exposure.^7^ Gender may also intersect with disability, ethnicity and other sources of social disempowerment to foster unequal health outcomes.^8^ Mobility restrictions, a lack of access to decision‐making power, lower literacy rates, discriminatory attributes of communities and healthcare providers due to a lack of training and awareness to the specific health needs and challenges of women and girls are just a few of these disparities.^9^

NTDs such as schistosomiasis, trachoma, hydrocele and kalazar, put men at an enhanced risk of disability and stigma, preventing them from performing their professional and social obligations as the family's breadwinner. However, for biological and cultural reasons, these NTDs disproportionately impact women and girls.^7^ As a result, long‐term health consequences such as neonatal mortality, infertility, blindness, and organ damage, among others, are unavoidable among women. An estimated 10 million women in Africa suffer from urinary schistosomiasis and anemia during pregnancy.^10^ Women in NTD‐affected areas have a higher risk of anemia due to hookworm infestation.^11^ Regarding household tasks like laundry, exposure of women to contaminated water sources like streams, rivers, and so forth, may cause schistosoma infestation in the urinary bladder, which may eventually lead to urogenital cancer.

Women are four times more likely than men to have lifelong blindness from trachoma because they spend more time with youngsters who have had the disease more than once.^12^ Women have also historically had a higher chance of acquiring podoconiosis due to cultural tradition and the availability of shoes that are perceived as being more suited for men.^13^, ^14^ There also exist an underreporting and underestimation of NTDs (in schistosomiasis and lymphatic filariasis). The rationale is that to diagnose this illness, women may need to follow culturally taboo or improper procedures including submitting urine or stool samples that allow for an intimate physical inspection.^15^ Financial and time restrictions are another factor that contributes to the increased outcome of NTDs in women. This is a result of the social obligation placed on women to care for the home. This restricts their options to employment and financial independence. The ability to make an income may be compromised in some situations because they are forced to automatically renounce their employment position.^7^

## 3 GENDER EXCLUSION IN PREVENTION AND CONTROL PROGRAMME IN AFRICA

In NTD programs, there are instances of both men and women being left behind. For instance, due to social stigmatizing attitudes, internalized stigmatizing attitudes, and the gender insensitivity of leprosy services, women with leprosy often receive treatment later than males.^16^, ^17^ These characteristics also indicate that some leprous women have not yet been detected at all.^17^ Women's health outcomes are typically the focus of gendered analyses of NTD outcomes for individuals, with less attention paid to men's and nonbinary people's illness experiences. For women, the biological effects of NTD illnesses such leishmaniasis, schistosomiasis, and trachoma are better characterized.^16^ It is worthy of note that some diseases have gender‐specific disabilities that are known to be ignored in disease‐specific control methods, such as female genital schistosomiasis, which impacts women's sexual and reproductive health.^16^, ^17^

Considering job roles, the Act to End NTDs | West's analysis of the training data from 11 countries showed that women's roles in human resources for health for NTDs, were (in the majority of cases) markedly less common than those of men, with the percentages of women trained for these roles frequently hovering in the teens.^18^ Benin (approximately 20% from 2015 to 2017), Burkina Faso (below 30% for all 4 years), Guinea (30% on average), Mali (26%), Niger (21%), Sierra Leone (30% on average), and Togo were among the countries with the lowest proportion of female CDDs trained (21% on average).^18^ To further unveil this inequality, the WHO's Integrating a Gender, Equity, and Human Rights Focus into National Programming on Preventative Chemotherapy and Transmission Control for NTDs project from 2016 to 2019, acknowledged that the selection of community drug distributors were made primarily by and for men, with the assertion that women were too frail to assume the role.^19^ Many women will even when included, work in unpaid roles due to the type and quality of work that is available to them, reinforcing their disadvantageous economic condition and financial responsibilities.

Additionally, the absence of gender consideration in NTD programming is demonstrated by the paucity of sex‐disaggregated data in the NTD literature, which suggests that planners and implementers frequently are unable to monitor gender equity.^8^ Moreso, despite the fact that these data must be collected, it is unclear how much of them are actually reviewed and used to guide programmatic and policy decisions.^20^ Gender norms indeed play a role in these exclusions. Cultural and sociological diversity in Africa affects how people view and accept drug distributors.^20^ As a result, unfavorable male‐to‐female ratios among front‐line employees may be intended to reflect regional sociocultural circumstances. For instance, in response to religious and cultural standards, Nigeria has strategically developed various regional methods for drug distributors: in the north, men cannot enter a house if a woman is alone, therefore having more female drug distributors is required.^15^, ^20^

## 4 RECOMMENDATIONS

To foster gender inclusion and equity, solutions should be considered across multiple levels encompassing community, social, programmatic, and research domains. It is imperative to comprehensively address the impact of NTDs on health and well‐being in Africa through a gender equity lens. This approach aims to attain equal life outcomes for both men and women, acknowledging their diverse needs and interests, with the overarching goal of redistributing power and resources. By adopting a gender lens, we can effectively tackle gender‐specific NTD exposures, the underlying causes of socioeconomic disparities, and other gender dynamics that impede the efficient delivery of NTD programs at local and national scales.

Notably, caregiving responsibilities for young children and family members afflicted with NTDs should not solely fall on women; instead, a gender‐inclusive perspective is essential for both women and men. Moreover, targeted initiatives should be established to institute awareness campaigns and health promotions concerning NTDs, facilitating early disease treatment and prevention of lifelong disabilities for both genders. The establishment of gender norms influencing the division of labor between males and females, in conjunction with factors like age and socioeconomic status, can impact NTD exposure risks. Health promotion campaigns should encompass participation from both genders. Implementing community empowerment programs becomes paramount in enhancing women's autonomy to seek healthcare. This will effectively contribute to addressing SDG #5—women's empowerment—which intersects with SDG #3—universal health and well‐being.

Balancing the empowerment of women and girls in the NTD front‐line workforce without exposing them to heightened risks or exploitation is crucial. Expanding job roles within NTD programs for women with prior experiences of the diseases can help minimize stigmatization and discrimination, fostering social acceptance. Capacitating women within the health facility workforce to administer NTD interventions is essential. At the community level, women should be provided more opportunities to engage in community‐based services such as drug distribution. This can be achieved by recruiting and training local women as healthcare workers, including community health workers and midwives. These individuals are often more culturally accepted and can access remote areas where healthcare facilities are lacking. Schoolgirls should also be actively engaged in school‐based sensitization, awareness, and promotion activities targeting NTD control. Health systems must increase their support for and engagement with women in NTD programs, ensuring their contributions are valued, acknowledged, and integrated not only at the community level but also on a global scale.

For a comprehensive understanding and enhancement of gender equity in the health workforce, collecting sex‐disaggregated data serves as a pivotal starting point. Researchers should meticulously design data collection tools capable of capturing, analyzing, and interpreting gender‐based data and indicators. Gendered analyses of NTD outcomes should focus on women's health effects. Systematically collecting and analyzing sex‐disaggregated data on NTD prevalence, treatments, and outcomes will aid in identifying and addressing barriers and opportunities for diverse groups to access NTD prevention and treatment services.

In addition, securing funding and establishing partnerships, including public‐private collaborations, becomes indispensable. Donors, partners, community groups, civil society, nongovernmental organizations, and various women‐led initiatives should collaborate and share insights to advance gender equality. Through pooling resources and expertize, these partnerships can drive the advancement of gender‐inclusive strategies to combat NTDs. Achieving sustainable progress in NTD control and elimination necessitates equitable interventions and coverage. Equity forms the foundation for fostering multi‐sectoral actions and creating measures that ensure inclusion in the Universal Health Coverage framework.

## 5 CONCLUSION

In summary, achieving gender‐equitable access to NTD programs in Africa requires adopting a gender equity lens, empowering women through community engagement, and fostering public‐private collaborations focused on gender equality. All actors are needed to be engaged to ensure comprehensive and effective improvement in gender equity in NTD management. Considering the societal roles assumed by men and women in Africa, healthcare policies should acknowledge and leverage this understanding to successfully cater to all segments of the population. Funders and implementers can play a role in designing appropriate gender‐sensitive programs, cultural leaders can play a role in changing the gender biased roles which impose more burden on women, and academics can design research tools that allow for proper analysis, interpretation and understanding of data at gender level. These steps will contribute to breaking the cycle of poverty and promoting universal health coverage in the region.

## AUTHOR CONTRIBUTIONS

**Deborah Oluwaseun Shomuyiwa**: Conceptualization; writing—original draft; writing—review and editing. **Nsikakabasi Samuel George**: Conceptualization; data curation; writing—original draft; writing—review and editing. **Blessing Abai Sunday**: Writing—original draft; writing—review and editing. **Faith O. Omotayo**: Writing—original draft; writing—review and editing. **Martha Mwaba**: Writing—original draft; writing—review and editing. **Success Chekwubechukwu David**: Writing—original draft; writing—review and editing. **Maxencia Nabiryo**: Writing—original draft; writing—review and editing. **Yonah Yangaza**: Writing—original draft; writing—review and editing.

## CONFLICT OF INTEREST STATEMENT

The authors declare no conflict of interest.

## TRANSPARENCY STATEMENT

The lead author Martha Mwaba affirms that this manuscript is an honest, accurate, and transparent account of the study being reported; that no important aspects of the study have been omitted; and that any discrepancies from the study as planned (and, if relevant, registered) have been explained.

## Data Availability

Data available on request from the authors.
